# Supplementary material for: Linear and categorical coding units in the mouse gustatory cortex drive population dynamics and behavior in taste decision-making
Source: eLife. 2026 Jun 16;14:RP109313. doi: 10.7554/eLife.109313 (PMC13271740; doi:10.7554/eLife.109313)
Supplement: Peer review history

# Editor report: eLife Assessment

**DOI:** 10.7554/eLife.109313.3.sa0

**Authors:** Thorsten Kahnt

**Affiliations:** National Institute on Drug Abuse Intramural Research Program United States

**Keywords:** Compelling, Important

This **important** work advances our understanding of the single neuron coding types in the mouse gustatory cortex and the functional roles of these neurons for perceptual decision-making. The conclusions are based on **compelling** evidence from rigorous behavioral experiments, high-density electrophysiology, sophisticated data analysis, and neural network modeling with in silico perturbations of functionally-identified units. This work will be of broad interest to systems neuroscientists.

# Referee report: Reviewer #1 (Public review)

**DOI:** 10.7554/eLife.109313.3.sa1

The manuscript provides several important findings that advance our current knowledge about the function of the gustatory cortex (GC). The authors used high density electrophysiology to record neural activity during a sucrose/NaCl mixture discrimination task. They observed population-based activity capable of representing different mixtures in a linear fashion during the initial stimulus sampling period as well as representing the behavioral decision (i.e., lick left or right) at a later time point. Analyzing this data at the single neuron level, they observed functional subpopulations capable of encoding the specific mixture (e.g., 45/55), tastant (e.g., sucrose), and behavioral choice (e.g., lick left). To test the functional consequences of these subpopulations, they built a recurrent neural network model in order to "silence" specific functional subpopulations of GC neurons. The virtual ablation of these functional subpopulations altered virtual behavioral performance in a manner predicted by the subpopulation's presumed contribution.

Strengths:

Building a recurrent neural network model of the gustatory cortex allows the impact of the temporal sequence of functionally identifiable populations of neurons to be tested in a manner not otherwise possible. Specifically, the author's model links neural activity at the single neuron and population level with perceptual ability. The electrophysiology methods and analyses used to shape the network model are appropriate. Overall, the conclusions of the manuscript are well supported.

Weaknesses:

One minor weakness is the mismatch between the neural analyses and behavioral data. Neural analyses (i.e. population activity trajectories) indicate a separation of the neural activity associated with each mixture. Given this analysis, one might expect the psychometric curve to have a significantly steeper slope. One potential explanation is the concentration of the stimuli utilized in the mixture discrimination task. The authors utilize equivalent concentrations, rather than intensity matched concentrations. In this case, a single stimulus can (theoretically) dominant the perception of a mixture resulting in a biased behavioral response despite accurate concentration coding. Given the difficulty of iso-intensity matching concentrations, this concern is not paramount.

# Referee report: Reviewer #2 (Public review)

**DOI:** 10.7554/eLife.109313.3.sa2

Lang et al. investigate the contribution of individual neuronal encoding of specific task features to population dynamics and behavior. Using a taste based decision-making behavioral task with electrophysiology from the mouse gustatory cortex and computational modeling, the authors reveal that neurons encoding sensory, perceptual, and decision-related information with linear and categorical patterns are essential for driving neural population dynamics and behavioral performance. Their findings suggest that individual linear and categorical coding units have a significant role in cortical dynamics and perceptual decision-making behavior.

Overall, the experimental and analytical work is of very high quality, and the findings are of great interest to the taste coding field, as well as to the broader systems neuroscience field.

I initially had some suggestions for further analyses to clarify the contribution of constrained and unconstrained units. In the revised version, the authors have performed all the suggested analyses, further strengthening their conclusions.

# Referee report: Reviewer #3 (Public review)

**DOI:** 10.7554/eLife.109313.3.sa3

Primary taste cortex neurons show a variety of dynamic response profiles during taste decision making tasks, reflecting both sensory and decision variables. In the present study, Lang et al., set out to determine how neurons with distinct response profiles contribute to perceptual decisions about taste stimuli.

The methods with regard to the behavioral task and electrophysiological recordings/data analysis are straightforward, solid and appropriate. The computational model is presented in a clear and conceptually intuitive manner, although the details are outside of my area of expertise.

The experimental design features a simple 2-alternative forced choice task that yielded clear psychometric curves across a range of stimuli. In vivo recordings were performed using neuropixels and yielded an appropriate sample of single neuron responses. The strength of the model lies in the fact that it consists of single neurons whose response profiles mimic those recorded in vivo, and allows neuron-selective manipulation.

By virtually lesioning specific subsets of neurons in the network, the authors demonstrate that a relatively small populations of neurons with specific tuning profiles were sufficient to produce the observed neural dynamics and behavioral responses. This effect was selective as lesioning other responsive neurons did not affect overall response dynamics or performance.

These findings provide new insight into the relation between the response profiles of single neurons in sensory cortex, their population-level activity dynamics, and the perceptual decisions they inform.

The approach is particularly innovative as it uses computational modeling to target functionally-defined "cell types", which cannot necessarily be targeted by more conventional genetic approaches.

# Author comment: Author response

**DOI:** 10.7554/eLife.109313.3.sa4

**Authors:** Liam Lang, Camelia Yuejiao Zheng, Jennifer M Blackwell, Giancarlo La Camera, Alfredo Fontanini

**Affiliations:** Stony Brook University Stony Brook United States; Stony Brook University Stony Brook United States; Stony Brook University Stony Brook United States; Stony Brook University Stony Brook United States; Stony Brook University Stony Brook United States

The following is the authors’ response to the original reviews.

**Reviewer #1 (Public review):**

This manuscript provides several important findings that advance our current knowledge about the function of the gustatory cortex (GC). The authors used high-density electrophysiology to record neural activity during a sucrose/NaCl mixture discrimination task. They observed population-based activity capable of representing different mixtures in a linear fashion during the initial stimulus sampling period, as well as representing the behavioral decision (i.e., lick left or right) at a later time point. Analyzing this data at the single neuron level, they observed functional subpopulations capable of encoding the specific mixture (e.g., 45/55), tastant (e.g., sucrose), and behavioral choice (e.g., lick left). To test the functional consequences of these subpopulations, they built a recurrent neural network model in order to "silence" specific functional subpopulations of GC neurons. The virtual ablation of these functional subpopulations altered virtual behavioral performance in a manner predicted by the subpopulation's presumed contribution.

Strengths:

Building a recurrent neural network model of the gustatory cortex allows the impact of the temporal sequence of functionally identifiable populations of neurons to be tested in a manner not otherwise possible. Specifically, the author's model links neural activity at the single neuron and population level with perceptual ability. The electrophysiology methods and analyses used to shape the network model are appropriate. Overall, the conclusions of the manuscript are well supported.

Weaknesses:

One potential concern is the apparent mismatch between the neural and behavioral data. Neural analyses indicate a clear separation of the activity associated with each mixture that is independent of the animal's ultimate choice. This would seemingly indicate that the animals are making errors despite correctly encoding the stimulus. Based solely on the neural data, one would expect the psychometric curve to be more "step-like" with a significantly steeper slope. One potential explanation for this observation is the concentration of the stimuli utilized in the mixture discrimination task. The authors utilize equivalent concentrations, rather than intensity-matched concentrations. In this case, a single stimulus can (theoretically) dominate the perception of a mixture, resulting in a biased behavioral response despite accurate concentration coding at the single neuron level. Given the difficulty of isointensity matching concentrations, this concern is not paramount. However, the apparent mismatch between the neural and behavioral data should be acknowledged/addressed in the text.

We thank the Reviewer for the insightful comments and thoughtful suggestions. Our electrophysiological recordings show that GC dynamically encodes stimulus concentration of mixture elements, dominant perceptual quality, and decisions of directional lick. With regard to the encoding of mixtures, the clear separation of activity associated with each mixture (Figure 3) is present at a trial-averaged pseudo-population level, and average activities associated with more similar, intermediate mixtures are closer to each other in this space. At a single trial level activities evoked by similar, intermediate mixtures are much harder to separate. This increased similarity can lead to behavioral errors resulting from either incorrect encoding of the stimulus or from the inability to interpret the stimulus to guide the correct decision. The psychometric function, which shows that more distinct stimuli (100/0 vs 0/100) lead to fewer mistakes than more ambiguous, intermediate mixtures (55/45 vs 55/45), is consistent with the increased ambiguity of responses to intermediate mixtures.

The Reviewer is correct that there could be a slight mismatch in the perceived intensity of the mixture components. This mismatch could be the reason for the slight asymmetry in our psychometric function (Figure 1B). However, it is not uncommon for mice in these 2AC tasks to also have a motor laterality bias in their responses that manifests itself for the more ambiguous stimuli. We chose not to model this bias given its subtlety and its unknown origin. Rather, we chose to model an ideal scenario in which stimuli have matched intensity and no motor bias exists. In the revised manuscript we discuss this issue.

**Reviewer #1 (Recommendations for the authors):**

(1) The apparent mismatch between neural and behavioral data. I am providing more details in this section to hopefully better illustrate my concern.

(a) Based on the author's psychometric curve, sucrose appears to be a more salient signal causing the behavior to be shifted (e.g., a 50/50 mixture results in a >60% predicted behavioral performance). If both sucrose and salt were intensity-matched, a 50/50 mixture should result in a behavioral performance near 50%. The increased salience of sucrose could cause the animals to have lower overall performance despite accurate neural encoding. Alternatively, certain animals could display a strong side bias, skewing the data slightly. These issues have seemingly been fixed in the model data, which displays a more balanced psychometric curve. Accordingly, the model data seemingly displays a larger shift in error trials as compared to correct trials (Figure 6A).

The reviewer is correct in observing that the average experimental psychometric curve in Figure 1B shows a slight shift in favor of the sucrose side with a 50/50 mixture. We fit psychometric curves to each session and the mean value of P(Sucrose choice | Stimulus = 50/50) across sessions was significantly different from 0.5 (one-sample t-test, p = 0.003), with 5 probabilities below 0.5 and 18 above it.

This slight bias could be attributed to a slight mismatch in the perceived intensity of the mixture components and/or lateral motor biases. In any case, it is subtle and its origins were not a focus of this study.

Models were not trained to match the animals’ psychometric curves, but rather to choose correctly in an ideal scenario where stimuli have matched intensities. This explains why the model simulations lack the bias observed in animal behavior data.

We do not believe that there is a mismatch between the experimental behavioral and neural data, as trial-averaged pseudo-population trajectories are farther in neural space for more discriminable stimuli and closer in neural space for more similar stimuli, consistent with behavioral performance that is high for more discriminable stimuli and low for more similar stimuli. Moreover, as the model also shows, a clear separation of trial-averaged trajectories still results in a sigmoidal performance function for trial-to-trial behavior.

Finally, subtle behavioral biases would not necessarily be expected to appear in our dPCA analyses since we used this technique to find a single axis that best separates all stimuli conditions regardless of choice when the pseudo-population data are projected upon it. Additional modes of activity that explain less overall variance might better reflect biases.

(b) Although I am not an expert at these analyses, I wonder whether the elevated bump (i.e., >0) in Figure 3C of the 55/45 mixture that occurs early in the stimulus presentation further supports the hypothesis mentioned above and could indicate an early signal of salience/increased intensity?

The reviewer is correct that the 55/45 trajectory features a brief positive wave right after stimulus delivery before going negative. While this may be related to stimuli not being explicitly balanced for intensity, it could also reflect a signal related to ambiguity or balanced mixtures. We are hesitant to interpret this positive deflection as conclusive evidence of a bias in neural activity, given its short duration and the natural variability of neural signals.

(2) The increase in step-perception neurons after the decision period is confusing (Figure 4C). The text states (line 246) "the analysis reveals a small and time-invariant proportion of step-perception neurons". However, the proportion doubles after the decision-making process, which is seemingly a significant change. Why does this occur? This observation is noticeably missing from the network data. Could it be attributed to a mislabeling of "step-choice" neurons, given the correlation between the left/right decision and sweet/salty? Either way, it is very noticeable and should be addressed.

We cannot be sure of the reason for the increase in step-perception neurons after decisions. One possibility is that they are acting as feedback for learning, encoding the percept to compare with choice and outcome to improve performance. The model, which presumably learns the task differently from the animals, does not seem to leverage this signal for its own learning. We have modified the text, now referring to a “small but consistently present proportion” of step-perception neurons, and included this proposed explanation in the Discussion.

(3) Optional: I think the authors are missing an opportunity to analyze the temporal aspect of this multiplex code using their network-based modeling approach. A significant proportion of neurons fall into different categories (i.e., step-perception/linear, etc.) at different time points. However, the virtual ablation experiments remove any neuron that falls into one of these categories at any time. By limiting the cell-specific virtual ablation to specific time windows, you could (I think) provide stronger evidence for the temporal sequence of the encoding of these perceptual aspects.

This was an excellent suggestion for an additional modeling experiment, so we performed it. A new supplemental figure (Figure S8) and additional text in the revised manuscript showcase the results. In summary:

In terms of behavioral results, ablating the linear coding units in the beginning (that is, silencing all units that are labeled linear in any bin within the first 1.2 s after stimulus onset for the entirety of the 1.2 s) significantly reduces performance, as does ablating the step-perception or step-choice coding units at the end (1.2 s prior to choice). The remaining combinations of coding type and timing of the ablation do not affect performance.

Regarding the dynamics of coding types (compare Figure 7A), stimulus coding activity was significantly blunted only by ablating the linear coding units in the beginning, whereas choice coding activity was diminished by ablating the choice coding units at the end or by ablating the linear coding units at either the beginning or the end.

**Reviewer #2 (Public review):**

Lang et al. investigate the contribution of individual neuronal encoding of specific task features to population dynamics and behavior. Using a taste-based decision-making behavioral task with electrophysiology from the mouse gustatory cortex and computational modeling, the authors reveal that neurons encoding sensory, perceptual, and decision-related information with linear and categorical patterns are essential for driving neural population dynamics and behavioral performance. Their findings suggest that individual linear and categorical coding units have a significant role in cortical dynamics and perceptual decision-making behavior.

Overall, the experimental and analytical work is of very high quality, and the findings are of great interest to the taste coding field, as well as to the broader systems neuroscience field.

I have a couple of suggestions to further enhance the authors' important conclusions:

My main comment is the distinction between constrained and unconstrained units. The authors train a small percentage of units to match the real neural data (constrained units), and then find some unconstrained units that are similar to the real neural data and some that are not. As far as I could tell, the relative fraction of constrained and unconstrained units in the trained RNN is not reported; I assume the constrained ones are a much smaller population, but this is unclear. The selection of different groups of neurons for the RNN ablation experiments appears to be based on their response profiles only. Therefore, if I understood correctly, both constrained and unconstrained units are ablated together for a given response category (e.g., linear or step-perception). It would be useful, therefore, to separately compare the effects of constrained vs. unconstrained RNN units.

We thank the Reviewer for the constructive feedback. The Reviewer is correct that ablations were carried out with respect to response categories only and included both constrained and unconstrained units.

The ratio of total units to constrained units was fixed at 5.88, thus constrained units were ~17% of the network and unconstrained units were ~83%. This value is specified in the Methods (RNN: Components and dynamics), but we have reported it in the Results of the revised manuscript for clarity.

We have also edited the Methods because they wrongly stated that the ratio of unconstrained (rather than total) units to constrained units was 5.88.

Specifically:

(1) For the analyses in the initial version of the manuscript, the authors should specify how many units in each ablation category are constrained and unconstrained.

In the revised manuscript, we have specified the fractions of constrained and unconstrained units within each response category. For convenience, they are reported here: linear = 194 constrained and 691 unconstrained units; step-perception = 147 constrained and 840 unconstrained units; step-choice = 129 constrained and 814 unconstrained units; “other” = 353 constrained and 1739 unconstrained units.

(2) The authors should repeat Figure 6, but only for unconstrained units to test how much of the effects in the initial version of Figure 6 are driven by constrained vs. unconstrained RNN units.

In the revised version we have included two additional supplemental figures (Figures S5-6) where the analyses of Figure 6 are carried out separately for constrained and unconstrained units. In short, the results for the constrained units strongly resemble those for the experimental data, while the results for the unconstrained units strongly resemble those for all model units.

(3) The authors should repeat Figure 7, but performing ablations separately on the constrained and unconstrained units to examine how the network behaves in each case and the resulting "behavioral" effect.

The revised version includes a supplemental figure (Figure S7) with the results of these additional ablation simulations.

In summary:

In terms of behavioral performance, the prior results showing that ablating linear, step-perception, or step-choice units significantly impairs performance, while ablating “other” has no significant effect, hold even if ablation is restricted to only constrained or only unconstrained units. There is a significant main effect of constrained vs unconstrained; on average, ablating the unconstrained population impairs performance more, most likely due to their larger population size.

In terms of dynamics, to impair stimulus coding by ablating step-choice units, you must ablate them all; to impair stimulus coding by ablating linear or step-perception units, however, ablating just the unconstrained ones suffices. As before, ablating linear, step-perception, or step-choice units significantly impairs choice coding activity, while ablating “other” units does not; these results hold even if ablation is restricted to only constrained or only unconstrained units. Finally, there is again a significant main effect of constrained vs unconstrained; on average, ablating the unconstrained population impairs dynamics more, most likely due to the larger population size.

**Reviewer #2 (Recommendations for the authors):**

(1) In addition to panel 5B, it would be informative to show data from individual mice and the corresponding RNNs trained on each mouse, to assess how closely they match. If available, including one representative example of a good match and one of a less accurate match would help the reader get a better sense of the data.

Figure 5B shows the average behavioral performance of the model. Individual models were not trained directly on the psychometric curves of experimental sessions; they were trained to perform the task correctly. After successful training, model simulations were run with input noise to be able to produce a sigmoidal psychometric curve. However, although the input noise was tuned to capture the overall correct rate of the corresponding experimental session, we did not attempt to match the details of the psychometric curve. See also the next reply.

(2) In addition to panel 5C, it would be useful to add examples of experimentally observed PSTHs and the corresponding activity trajectory for the units in the RNN trained to match them, for all the other coding patterns (step-perception and step-choice).

We note that the PSTH in 5C is not an example of a linear coding unit as the Reviewer implies, but simply one with a good fit, and here the model’s output was produced in the absence of input noise. In order to classify step-perception and step-choice responses one needs error trials, but the model was trained without this input noise that induces errors (and produces a sigmoidal psychometric function) to match experimental PSTHs from correct trials only. Post-training simulations were then run with input noise to induce error trials, and model unit response profiles were classified based on this. However, there is no guarantee that error trials in the model match the error trials in the experiment; therefore, step-perception and step-choice units in the model may or may not be step-perception and step-choice units in the data. Despite this limitation, the revised manuscript includes additional examples, in Figure S2, of experimentally observed PSTHs and their corresponding model activity, to supplement Figure 5C and provide a better sense of the goodness-of-fit.

(3) Electrophysiological data in Figure 2 - It would be helpful to provide statistics on how many neurons change their activity in each session.

In the revised manuscript we have included across-session statistics for proportions of neurons that are taste-responsive and that show decision preparatory activity. We have also included tables (Tables S1 and S3) with the numbers of neurons that are taste-responsive and that show preparatory activity for each session in the experimental and model data.

(4) Peak auROC selection - How was the peak auROC selected? Selecting only one bin for the peak could be potentially problematic and may result in the incorrect identification of an outlier that does not faithfully represent the neuron's overall activity. The peak selection could instead be based on several consecutive bins showing a consistent trend. If this approach was already implemented, the authors should explicitly describe it in the Methods section.

Peak auROC was selected from a single bin (with average duration about 50ms). While it is true that this may result in outlier neurons that transiently prefer one stimulus strongly but more consistently prefer the other, we opted for a simple criterion to sort the neurons into two categories for visualization. Adopting more stringent criteria that consider multiple bins may result in neurons that cannot be placed in either category, and we wanted a way to examine the entire pseudo-population. Also, the entire auROC trace is visualized in the heatmap, so potential outliers are not hidden and can be assessed by eye.

**Reviewer #3 (Public review):**

Primary taste cortex neurons show a variety of dynamic response profiles during taste decision-making tasks, reflecting both sensory and decision variables. In the present study, Lang et al. set out to determine how neurons with distinct response profiles contribute to perceptual decisions about taste stimuli.

The methods, with reference to the behavioral task and electrophysiological recordings/data analysis, are straightforward, solid, and appropriate. The computational model is presented in a clear and conceptually intuitive manner, although the details are outside of my area of expertise.

The experimental design features a simple 2-alternative forced-choice design that yielded clear psychometric curves across a range of stimuli. In vivo recordings were performed using Neuropixels and yielded an appropriate sample of single neuron responses. The strength of the model lies in the fact that it consists of single neurons whose response profiles mimic those recorded in vivo, and allows neuron-selective manipulation.

By virtually lesioning specific subsets of neurons in the network, the authors demonstrate that a relatively small population of neurons with specific tuning profiles was sufficient to produce the observed neural dynamics and behavioral responses. This effect was selective as lesioning other responsive neurons did not affect overall response dynamics or performance.

These findings provide new insight into the relation between the response profiles of single neurons in sensory cortex, their population-level activity dynamics, and the perceptual decisions they inform.

The approach is particularly innovative as it uses computational modeling to target functionally-defined "cell types", which cannot necessarily be targeted by more conventional genetic approaches.

We thank the Reviewer for the positive assessment of our study.

**Reviewer #3 (Recommendations for the authors):**

(1) Introduction: I'm missing a clearly stated specific hypothesis and what is predicted on the basis of that hypothesis. What is the alternative?

The null hypothesis is that single neuron activity patterns, even when clearly structured, do not matter for population activity or behavior. Alternatively, they do matter for these phenomena, and our model supports the alternative hypothesis. We have made this hypothesis clearer in the Introduction.

(2) Discussion: Much of the text is a recap of the Introduction and Results sections. Please elaborate on the specific insights gained from the findings. The idea that tuned neurons in the sensory cortex are the basis for perception and perceptual decisions concerning the features being represented by those neurons is generally accepted. What the present study adds to this insight could be described more explicitly. On the other hand, the idea that small populations of tuned neurons are responsible for perception of taste/perceptual decisions about taste appears in contrast with previous accounts where stimulus features/decisions are reflected in correlated changes in activity across distributed populations of taste cortical neurons, including ones that are not necessarily tuned or even overtly responsive. How do the present findings relate to this idea?

This is a very good point about reconciling these findings with past ones that have focused on coordinated changes across ensembles of neurons, i.e., metastable dynamics of internal (hidden) states. There is a brief mention of metastability toward the end of the Discussion, but we agree it deserves elaboration.

This work does emphasize single unit activity, but in the context of, and as relevant to, population activity. We believe that the findings and frameworks of previous studies and those presented here are compatible rather than mutually exclusive. There is no reason why neurons with the coding patterns we studied here cannot coordinate with others to participate in the formation of different metastable states. The question of which—neurons with specific response profiles, or ensemble activity patterns that may involve these neurons?—is necessary and sufficient for producing perception and behavior during the mixture-based decision-making task is interesting but rather difficult to answer because of the single units’ contribution to both alternatives. One would need to utilize a manipulation that disrupts ensemble coordination without disrupting single unit activity to differentiate between them. We have made these points clearer in the Discussion.

(3) Results: RNNs were based on data from single sessions -- how many neurons of each tuning type were observed in each session? In particular, there were 23 sessions but only 25 neurons total tuned to choice, suggesting that modelled choice neurons were based on ~1 neuron.

The revised manuscript includes the session-by-session breakdown of response types for both experiment and model in two supplementary tables (Tables S2 and S4). We note that there are 25 neurons tuned to choice during the last 500 ms of the trial prior to decision, but 114 out of 626 neurons in total are tuned to choice in some time bin in the experimental data.

(4) Minor: Indicate the time windows used for analysis of stimulus sampling, delay, and choice on the figures.

The revised manuscript now includes the illustration of sampling and delay windows in Figure 2C-D, since we averaged the values over these windows for use in a 2-way ANOVA. All other figures either are associated with bin-by-bin analyses and have the first central and lateral licks (T and D) indicated, or have the time windows specified (e.g., Figure 4B, which uses [T, T + 0.5 s] and [D - 0.5 s, D]).
